# Supplementary material for: The DNA damage response pathway regulates the expression of the immune checkpoint CD47
Source: Commun Biol. 2023 Mar 7;6:245. doi: 10.1038/s42003-023-04615-6 (PMC9992352; doi:10.1038/s42003-023-04615-6)
Supplement: Supplementary file 2 — Supplementary Figures [file 42003_2023_4615_MOESM2_ESM.pdf]

SUPPLEMENTARY FIGURES

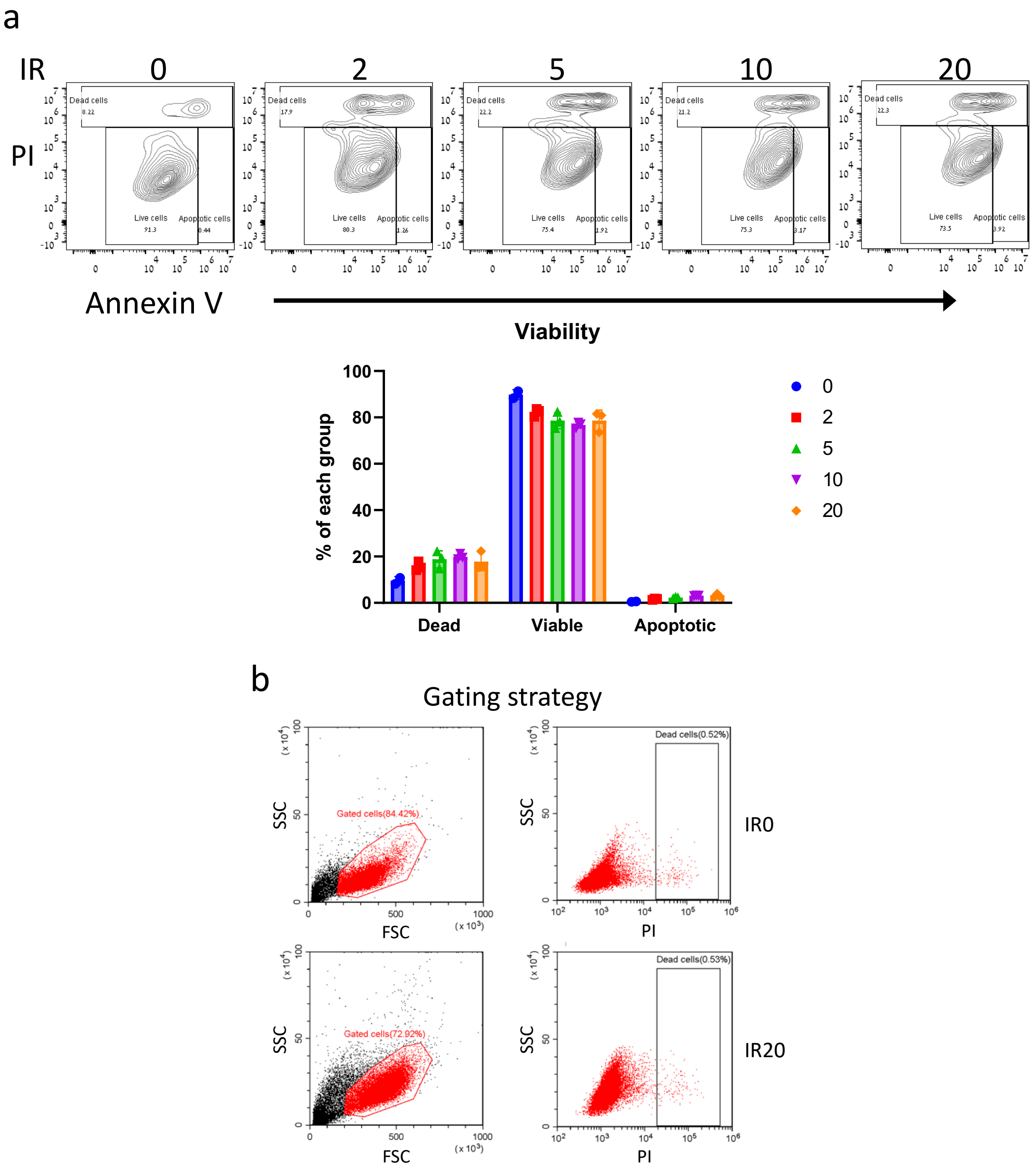

Figure S1: *Cell viability following* γ-irradiation
A. HEK-293T cells were irradiated at the indicated dose and 24h later cells were collected and stained with Annexin V-FITC and propidium iodide (PI). The percentage of dead, live and apoptotic cells are shown in the graph as an average (± STD) of four replicates in each group. A representative experiment of at two independent experiments is shown. B. The FSC/SSC gating strategy of live cells is shown.

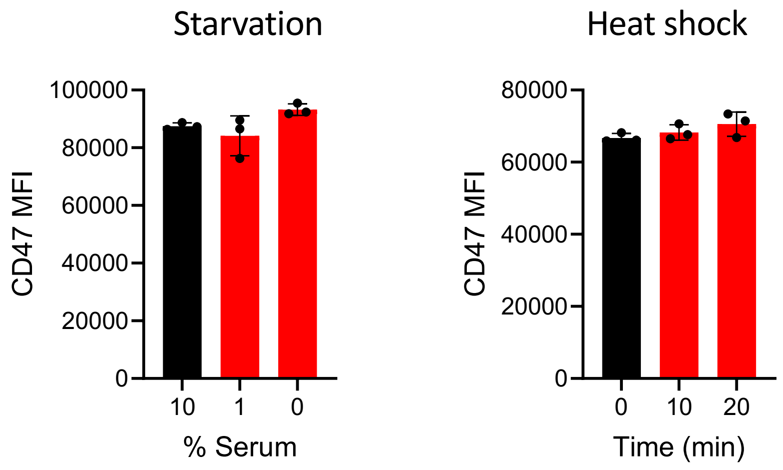

Figure S2: *Heat shock and serum starvation do not induce CD47 overexpression.* HEK-293T cells were incubated in 42°C for 10 and 20 minutes or incubated in medium containing 10, 1 or 0 percent serum for 24h. CD47 expression (MFI) on HEK-293T cells' surface24h after treatment is presnted. Graphs show an average (± STD) of four replicates in each group. A representative experiment of at least four independent experiments is shown. ns: non-significant.

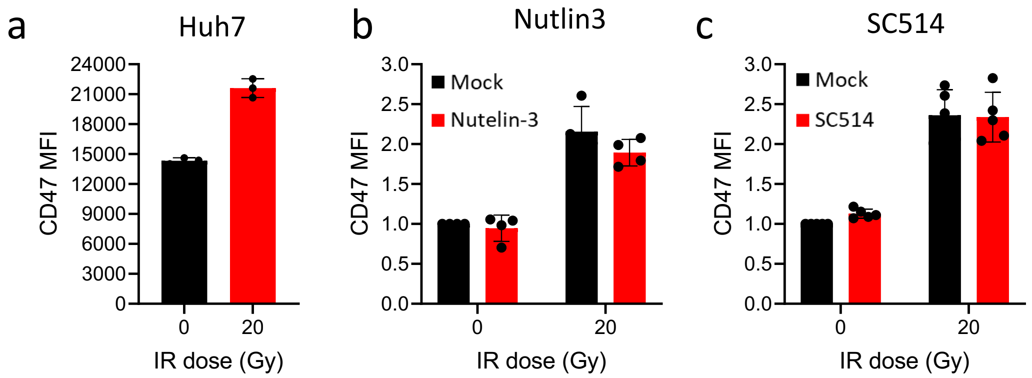

Figure S3: Overexpression of CD47 in response to γ-irradiation is not dependent on p53, NFkB pathways

A. CD47 expression in Huh7 cells following γ-irradiation. **B-C.** HEK-293T cells were treated with either nutelin-3 (**B**) or SC514 (**C**) and then exposed to γ-irradiation. Graphs depict CD47 expression (MFI). Graph shows an average (± STD) of four independent experiments. ns: non-significant; ** p<0.005.

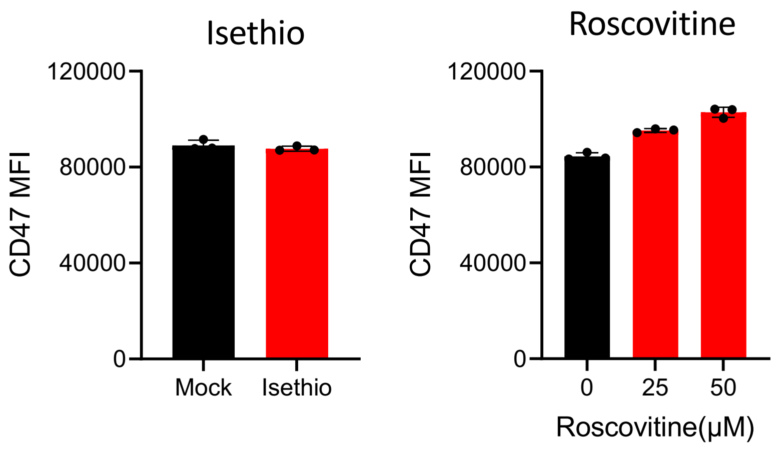

Figure S4: *Cell cycle arrest does not induce CD47 overexpression*.
HEK-293T cells were incubated with the indicated CDK inhibitors for 24h and CD47 expression was determined. Graphs show an average (± STD) of four replicates in each group. One of at least four independent experiments is shown. ns: non-significant.

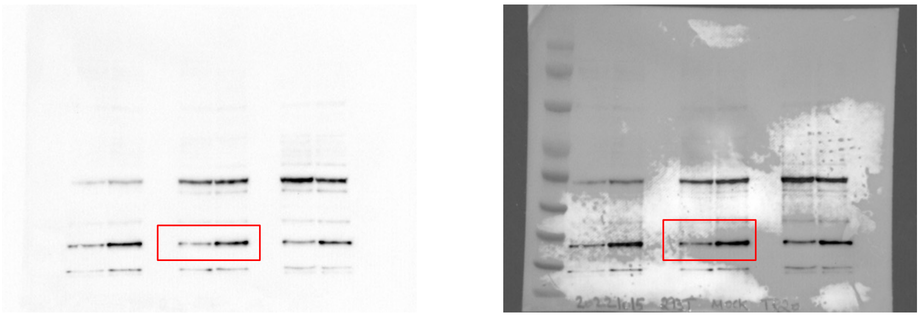

CD47

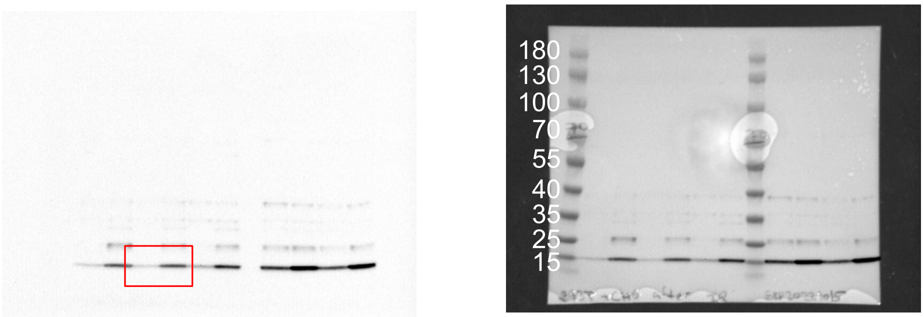

γH2AX

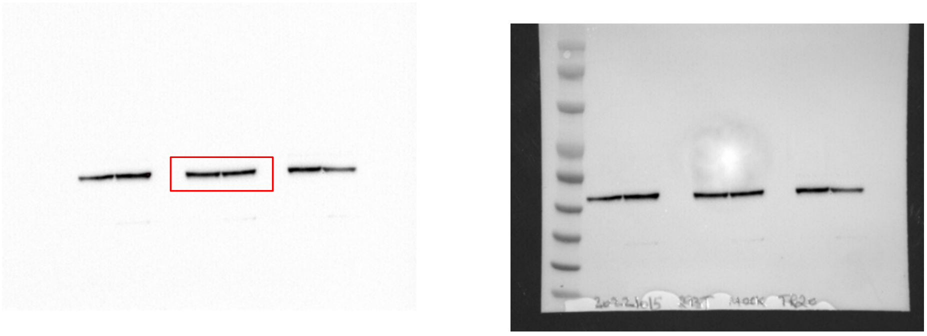

Actin

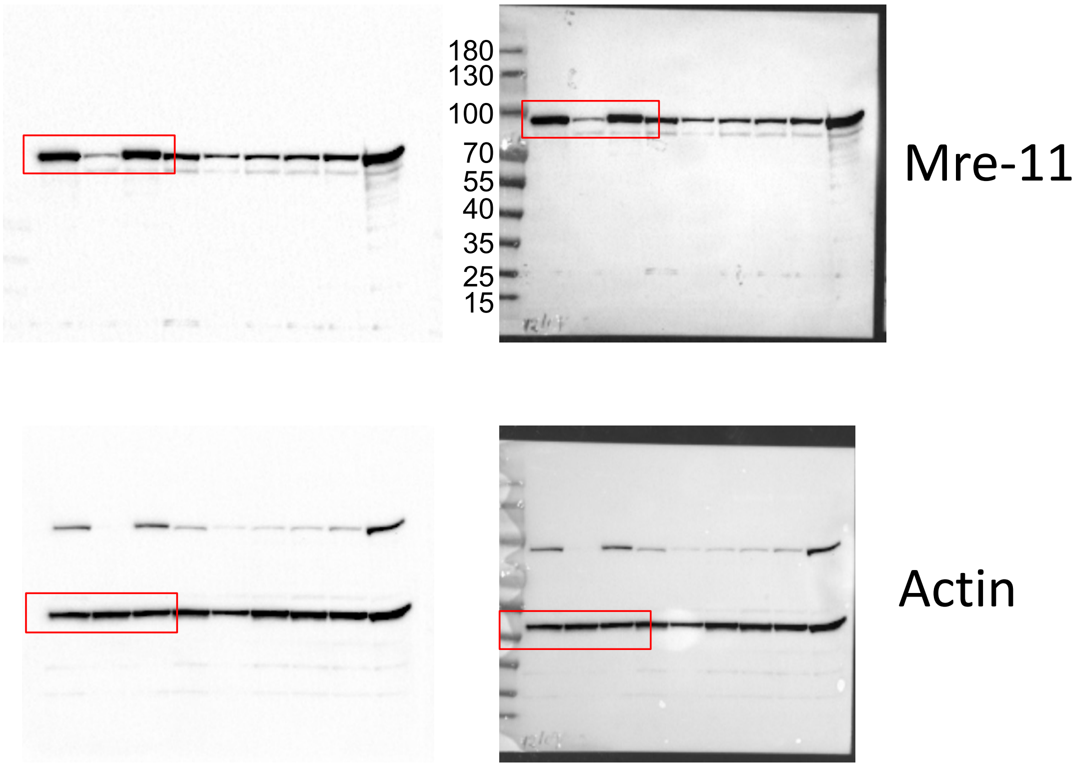

Figure S5: *Unprocessed scans of Western Blotting in figures 3 and 4*
